# A multi-level developmental approach to exploring individual differences in Down syndrome: genes, brain, behaviour, and environment

**DOI:** 10.1016/j.ridd.2020.103638

**Published:** 2020-09

**Authors:** Michael S.C. Thomas, Olatz Ojinaga Alfageme, Hana D’Souza, Prachi A. Patkee, Mary A. Rutherford, Kin Y. Mok, John Hardy, Annette Karmiloff-Smith, the LonDownS Consortium

**Affiliations:** a Centre for Brain and Cognitive Development, Birkbeck, University of London, London WC1E 7HX, United Kingdom; b Department of Psychology & Newnham College, University of Cambridge, Cambridge CB3 9DF, United Kingdom; c Centre for the Developing Brain, School of Biomedical Engineering and Imaging Sciences, King’s College London, St. Thomas’s Hospital, London, SE1 7EH, United Kingdom; d Department of Neurodegenerative Disease, Institute of Neurology, University College London, United Kingdom

**Keywords:** Down syndrome, individual differences, brain imaging, genetics, Alzheimer’s disease, vocabulary development, apolipoprotein *APOE* gene, socio-economic status

## Abstract

In this article, we focus on the causes of individual differences in Down syndrome (DS), exemplifying the multi-level, multi-method, lifespan developmental approach advocated by Karmiloff-Smith (1998, 2009, 2012, 2016). We evaluate the possibility of linking variations in infant and child development with variations in the (elevated) risk for Alzheimer’s disease (AD) in adults with DS. We review the theoretical basis for this argument, considering genetics, epigenetics, brain, behaviour and environment. In studies 1 and 2, we focus on variation in language development. We utilise data from the MacArthur-Bates Communicative Development Inventories (CDI; Fenson et al., 2007), and Mullen Scales of Early Learning (MSEL) receptive and productive language subscales (Mullen, 1995) from 84 infants and children with DS (mean age 2;3, range 0;7 to 5;3). As expected, there was developmental delay in both receptive and expressive vocabulary and wide individual differences. Study 1 examined the influence of an environmental measure (socio-economic status as measured by parental occupation) on the observed variability. SES did not predict a reliable amount of the variation. Study 2 examined the predictive power of a specific genetic measure (apolipoprotein *APOE* genotype) which modulates risk for AD in adulthood. There was no reliable effect of *APOE* genotype, though weak evidence that development was faster for the genotype conferring greater AD risk (ε4 carriers), consistent with recent observations in infant attention (D’Souza, Mason et al., 2020). Study 3 considered the concerted effect of the DS genotype on early brain development. We describe new magnetic resonance imaging methods for measuring prenatal and neonatal brain structure in DS (e.g., volumes of supratentorial brain, cortex, cerebellar volume; Patkee et al., 2019). We establish the methodological viability of linking differences in early brain structure to measures of infant cognitive development, measured by the MSEL, as a potential early marker of clinical relevance. Five case studies are presented as proof of concept, but these are as yet too few to discern a pattern.

## Highlights

• A consideration of the causes of individual differences in Down syndrome, at the level of genes, epigenetics, brain, and behaviour, linking potential differences in early development with elevated risk for Alzheimer’s disease.
• Evaluation of environmental (socioeconomic status) and genetic (chromosome 19 apolipoprotein *APOE* genotype, modulating risk for AD in adulthood) predictors of individual differences in early vocabulary development in a sample of 84 infants and young children with DS. Neither predictor accounted for significant amounts of variance, leaving the wide variability unexplained and likely arising from complex individual effects of the DS genotype. There was weak evidence that early development was faster for the *APOE* genotype conferring greater AD risk (ε4 carriers) consistent with recent observations in infant attention (D’Souza, Mason et al., 2020)
• Proof of principle that prenatal and neonatal magnetic resonance imaging methods may be used to test the predictive power of measures of early brain structure for variation in DS infant cognitive development. Early brain development represents the concerted effect of the DS genotype.
• The article argues for the importance of a multi-level, lifespan developmental approach to explore the origins of individual differences in DS cognition. It hypothesises that in this framework, dementia itself may be considered as a developmental disorder (Karmiloff-Smith et al., 2016).

## What this paper adds

• A consideration of the causes of individual differences in Down syndrome, at the level of genes, epigenetics, brain, and behaviour, linking potential differences in early development with elevated risk for Alzheimer’s disease.
• Evaluation of environmental (socioeconomic status) and genetic (chromosome 19 apolipoprotein *APOE* genotype, modulating risk for AD in adulthood) predictors of individual differences in early vocabulary development in a sample of 84 infants and young children with DS. Neither predictor accounted for significant amounts of variance, leaving the wide variability unexplained and likely arising from complex individual effects of the DS genotype.
• Proof of principle how prenatal and neonatal magnetic resonance imaging methods may be used to test the predictive power of measures of early brain structure for variation in DS infant cognitive development. Early brain development represents a marker of the concerted effect of the DS genotype.
• The article argues for the importance of a multi-level, multi-method lifespan developmental approach to explore the origins of individual differences in DS cognition. It hypothesises that in this framework, dementia itself may be considered as a developmental disorder (Karmiloff-Smith et al., 2016).

Down syndrome (DS), arising from trisomy of chromosome 21, is the most common genetic syndrome causing intellectual disability, with an incidence of between 1 in 750 and 1 in 1000 live births (Morris et al., 2012; Parker et al., 2010). Some characteristics of the syndrome are near universal, including a characteristic facial appearance, learning disability, muscle hypotonia, motor dysfunction, and Alzheimer’s disease neuropathology after 35 years of age (Antonarakis et al., 2004). However, there are large individual differences in the DS phenotype and severity of associated symptoms (Dykens & Hodapp, 2001; Karmiloff-Smith et al., 2016; Rachidi & Lopez, 2007). In this paper, we exemplify a multi-level approach to considering the causes of individual variation in the DS cognitive phenotype across the lifespan, and argue for linking variation in early development with dementia in later life within a common framework. In the first section, we consider the theoretical grounds for making this argument. In the second section, we demonstrate the individual differences in one cognitive phenotype, early vocabulary development, in a sample of 84 infants and toddlers with DS, seeking to explain some of the individual variability via a common environmental measure (family socioeconomic status). In the third section, we assess the contribution to variability in vocabulary development of a single genetic measure that confers risk for Alzheimer’s disease (apolipoprotein genotype). In the fourth section, we employ a set of case studies to demonstrate the viability of linking variation in prenatal and neonatal measures of brain structure in DS – as a broader outcome of many genetic factors – with variation in early cognitive development.

## 1 Linking variability in early cognitive development and adult dementia risk in Down syndrome – a common framework?

While general intellectual disability in DS is described as falling within the mild to severe range, there is marked individual variability; around 80% of individuals have moderate intellectual disability, but for some it is in the severe range and others have IQ scores falling within the normal range (Chapman & Hesketh, 2000; Pueschel, 1995; Roizen, 2007). The general profile includes particular difficulties in motor ability, auditory processing, verbal short-term memory, and expressive language, while there are relative strengths in visuospatial processing, receptive language, and some aspects of social functioning but again, this profile varies between individuals (Daunhauer & Fidler, 2011; Jarrold & Baddeley, 1997; Jarrold et al., 1999; Miller, 1999; Miller & Leddy, 1999; Pitcairn & Wishart, 1994; Vicari, 2006; Vicari & Carlesimo, 2006; Wishart & Johnston, 1990). For example, while social functioning is often a relative strength, with children forming interpersonal relationships in similar ways to typically developing (TD) peers (Freeman & Kasari, 2002), some children have deficits in this area even to the extent of co-morbid diagnoses of autism (Glennon et al., 2017). A recent study of early development in DS (D’Souza et al., 2020), which combined cross-sectional and longitudinal trajectories in the Mullen Scales of Early Learning (Mullen, 1995) from 6 months to 5 years, indicated that across several of the subtests (fine motor, visual reception, receptive language, expressive language), children with DS exhibited an initial profile similar to TD children of equivalent chronological age, but then diverged over developmental time. This pattern was especially pronounced for expressive language, which became an area of relative weakness due to a particularly slow pace of development. In contrast, the gross motor domain presented an area of relative weakness throughout the first five years of life. The authors again reported large variability between children; indeed, that the variability increased with age, suggesting diverging developmental trajectories within the DS group itself.

In adulthood, individuals with DS are at elevated risk for Alzheimer’s disease (AD). This is largely due to the fact that the amyloid precursor protein (*APP*) gene, which is implicated in the brain pathology of AD, lies on the triplicated chromosome 21. Reports of the prevalence of dementia between the ages of 30 and 39 years range between 0 to 33% of individuals with DS, between 40-49 years of age from 5.7-55%, and between 50-59 years from 4-55% (Head, Powell et al., 2012). Notably, even though AD neuropathology – that is, protein build-up in the brain – is present in virtually all adults with DS from the mid 30 s, there is a subset of individuals who do not appear to develop clinical signs of dementia, suggesting individual differences in possible protective or resilience factors (Head, Powell et al., 2012; Head, Silverman et al., 2012; Krinsky-McHale et al., 2008). Individual differences in DS are therefore a feature of both early development and ageing.

Is there any basis to believe that the causes of these respective individual differences could be linked? Karmiloff-Smith (1998) has argued that the process of development itself is the key to understanding developmental disorders. Deficits observable at any specific age should be viewed as the outcome of an atypical developmental process, to be understood at multiple levels of description, including genes, brains, cognition, behaviour, and environment. To understand the cause of cognitive deficits observed in childhood or adulthood, it is therefore necessary to trace them back to their origins in early infancy. In this perspective, ageing (healthy or otherwise) is part of continuous lifespan development, to be characterised in terms of mechanisms of change. More recently, Karmiloff-Smith (2016) argued that AD should not be merely considered a disease of ageing, but be thought of as a developmental disease. Even in the TD population, AD does not turn itself on overnight, it develops over time. In this sense, Karmiloff-Smith argued that studying DS might allow us to look at the development of AD in slow motion.

There is precedent to consider early developmental origins as relevant to the breakdown in behaviour in adulthood. For example, the age at which vocabulary items are acquired in early childhood is predictive of the loss of vocabulary items during dementia, with earlier acquired vocabulary more robust (Ellis, 2012). Although level of educational attainment appears to be predictive of resilience to cognitive decline in ageing, all of this explanatory power can be traced to the level of early cognitive development (correlated with subsequent educational attainment) (Berggren et al., 2018). Thus Berggren, Nilsson and Lövdén (2018) argue that education is only a relevant variable for understanding cognitive performance in older age because of the association between performance and education that is formed in early development. Tucker-Drob (2019) similarly argues that trajectories of child cognitive development determine peak levels of adult cognitive function from which aging-related cognitive declines occur. In both these examples, stronger early development predicts greater resilience to loss of skills in dementia, and conversely, weaker early development predicts greater vulnerability. The way that cognitive development assembles abilities accounts, to some extent, for the ways the abilities are disassembled under the processes of pathological ageing. While the ‘strength’ of development is a simplistic concept given the complex dynamics of developing systems, and stands in need of a biological basis, these types of link between development and dementia nevertheless suggest a research approach where we should seek genetic or environmental factors that explain variability in development and seek their predictive power in resilience to ageing, or vice versa.

The causes of individual differences in the severity of cognitive deficits in DS are complex and not well understood, with variability identified at multiple levels (Karmiloff-Smith et al., 2016). At the genetic level, 95% of cases arise from an additional copy of an entire copy of chromosome 21 (trisomy 21; Chapman & Hesketh, 2000), although the error in cell division can occur through different mechanisms. DS can also occur when only a segment of chromosome 21 has three copies (partial trisomy), or when only a proportion of cells have an additional copy (mosaicism) (Korbel et al., 2009; Papavassiliou et al., 2015). While mosaicism may be associated with differences in phenotypic severity (Korbel et al., 2009), the pattern is unclear, and variability is still apparent with full trisomy. To the extent that phenotypic variability is driven by genotypic variability, this implicates variation in the genetic information on chromosome 21 itself, or variation in the genes on other chromosomes with which those on chromosome 21 interact through complex biological networks. The complexity of interactions is illustrated by the fact that, although one might expect that expression levels of genes on chromosome 21 would be 1.5 times the normal level (3 copies of genes instead of 2), this is only true for some of the genes; others show normal expression and some intermediate levels, implicating feedback control mechanisms operating within these biological networks (Prandini et al., 2007; Ait Yahya-Graison et al., 2007). Variability may also arise through differential disruptions to epigenetic mechanisms controlling gene expression, with at least 11 genes on chromosome 21 involved in epigenetic mechanisms (Dekker et al., 2014).

At the brain level, magnetic resonance imaging (MRI) studies of adults have indicated differences in the size of macro-structures, with reduced cerebellar, hippocampal, and cortical volumes, and enlarged ventricles (Edgin, 2013; Strydom et al., 2004). Divergent developmental trajectories are apparent from 22 weeks gestation, where some but not all fetal brains show differences compared to controls (Baburamani et al., 2019; Patkee et al., 2019). Some but not all infants with DS show smaller dendritic arborisation and fewer synapses (e.g., Becker et al., 1986; Petit et al., 1984); and micro differences in connectivity are subsequently associated with reduced functional brain connectivity in infancy (Imai et al., 2014). Variability in health phenotypes may also indirectly contribute to differences in cognitive development, for example in cases of congenital heart defects altering blood oxygenation that impacts brain development (Baburamani et al., 2019), or sleep apnea compromising memory consolidation processes during sleep (Edgin et al., 2015).

At the environmental level, differences may exist in parental behaviour in response to a child with a disability (for example, in the sensitivity of parenting, or ability to scaffold the development of language or executive function skills), or in the parents’ mental health such as parental depression (see e.g., D’Souza, Lathan, Karmiloff-Smith & Mareschal, 2020); in the time and resources available to support the child in the home or school (for example, as indexed by differences in socio-economical status or neighbourhood demographics); and in the therapeutic resources available to the family (Cebula et al., 2010; Moore et al., 2002).

With respect to AD, the amyloid protein build up associated with three copies of the *APP* gene has been the main focus of the pathogenic process producing elevated risk of dementia in adults with DS. However, there have been links between this process and development. For example, using human induced pluripotent stem cells *in vitro*, Shi et al. (2012) showed growth of amyloid-β plaques (protein fragments snipped from an amyloid precursor protein) in cells grown from tissue from an infant with DS as young as 17 months of age. *In vivo* studies observed plaques in the brains of children as young as 8 years (Leverenz et al., 1998), although some DS brains do not show plaques until early adulthood, again indicating individual variation in this pathogenic process (Karmiloff-Smith et al., 2016). Several genes on chromosome 21 with increased expression in DS have also been shown to be relevant to the pathogenesis of AD, including *DYRK1A*, *RCAN1*, and *BACE2* (Abbassi et al., 2015; Lin et al., 2011; Myllykangas et al., 2005). One source of genetic variation outside of chromosome 21 that has received attention is the apolipoprotein (*APOE*) gene on chromosome 19. This is because it has been identified as a genetic risk factor for AD in the general population (Kim et al., 2009; Ritchie et al., 2019). The ε4 allele is an AD risk factor, the ε3 allele neutral, and the ε2 allele is protective; one copy of the ε4 allele increases risk of AD by 3-4-fold, two copies of ε4 by 12-16-fold, relative to possessing two copies of the ε3 allele, while there is a decreased risk associated with the ε2 allele (Smith et al., 2019). The risk factor potentially operates via the effect of *APOE* on amyloid-β metabolism. *APOE* genotype has also been found to modulate the age of onset and progression of AD in DS. Prasher et al. (2008) found that compared to carriers of the neutral ε3 allele, adult DS carriers of the ε4 allele had a 1.8 times greater risk of developing AD, an earlier onset (55 vs. 57 years) and a more rapid progression to death (4.2 years vs. 5.4 years). Notably, *APOE* variants have also been linked to processes of brain development. Dean et al. (2014) compared magnetic resonance imaging measurements of white matter myelin water fraction and grey matter volume in healthy infant carriers and non-carriers of the ε4 allele between 2 and 25 months, and found decreased rate of growth in myelin in mid and posterior brain regions, in areas preferentially affected by AD in adulthood. Smith, Ashford and Perfetti (2019) suggest that possession of the ε4 allele is associated with higher levels of synaptic macromolecular turnover, which may produce greater early plasticity but also stress basic cellular neuroplasticity mechanisms. For both *APP* and *APOE*, then, there are links between development and pathological ageing.

Our goal in the remainder of this paper is to exemplify a multilevel, multimethod lifespan developmental approach to studying individual variation in DS. We begin by focusing on a single cognitive domain, vocabulary development, because it is known to be sensitive to environmental factors in typical development, such as socioeconomic status (SES), and because it demonstrates notable individual variation across children with DS. In Section 2, we describe a study that assesses the extent to which environmental factors influence DS vocabulary development. In Section 3, we then assess whether a single genetic factor, *APOE* genotype, produces measurable influence on vocabulary development, in light of its known influence on cognitive decline in later life. In Section 4, we assess the viability of using prenatal and neonatal measures of brain structure as a marker for the broader outcome of many genetic factors acting on early brain development, now linking to a broader set of cognitive measures in infancy. In the final section, we consider different hypothetical relationships that may link early development and cognitive decline within the same explanatory framework.

## 2 Environmental factors influencing early vocabulary development in DS

Language is a domain of vulnerability in DS (Abbeduto et al., 2007; Chapman, 2006; Fidler et al., 2005). Receptive language is usually stronger than expressive language, speech intelligibility can be poor, and there are weaknesses in phonology and syntax (Martin et al., 2009). The acquisition of first words is delayed and subsequent growth is slow compared to TD expectations (Berglund et al., 2001). Once again, this is a domain of wide individual differences. At 36 months, Zampini and D’Odorico (2009) found that the lowest scoring child was non-verbal, while the highest fell in the normal range, producing 234 words. At 6-month follow-up, the former child was still non-verbal, the latter producing nearly 500 words. Another longitudinal study of a sample of children with DS from 2 to 7 years, which used an 18-month follow-up period, charted growth varying from around 400 additional words, to children who verbally expressed only a few more words or even expressed fewer words at follow up (Deckers et al., 2017). Poor speech intelligibility may affect productive language performance, which could explain some of the discrepancy between expressive and receptive language levels (Miller & Leddy, 1998) and children with DS vary in the extent to which they exploit gesture as an alternative modality to support their communication (te Kaat-van den Os et al., 2017).

Deckers et al. (2017) considered what factors predicted variation in vocabulary growth in their sample of 20 three-year-olds with DS. They observed that receptive vocabulary development was best predicted by adaptive level of functioning and earlier receptive vocabulary skills (demonstrating some stability in individual differences over time), while expressive vocabulary growth was best predicted by adaptive level of functioning, receptive vocabulary, level of maternal education, level of communicative intent of the child, attention skills, and phonological awareness. Partial correlations of maternal education to measures of expressive vocabulary, controlling for age, were between .47 and .56. In typical development, measures of SES have been correlated to individual differences in cognitive development, with language showing one of the strongest relationships (e.g., Farah et al., 2006). Farah et al. (2006) reported that SES accounted for up to 32% of the variance in a composite measure of language based on vocabulary and phonological processing. In a longitudinal study of 48 infants, Fernald, Marchman and Weisleder (2013) found that SES correlated with vocabulary size, measured via the MacArthur-Bates Communicative Development Inventories (Fenson et al., 2007), at .34 at 18 months of age and .29 at 24 months of age. We were therefore interested whether SES accounts for similar levels of variation in the vocabulary development of infants with DS.

In the following study, we began by considering the extent to which SES level, measured by highest parental occupation, predicted variance in DS receptive and expressive vocabulary scores in early childhood.

### 2.1 Method

#### 2.1.1 Participants

The sample comprised 84 infants and young children with DS (48 male, 36 female), recruited as part of the larger LonDownS study (see Startin et al., 2020). The children were aged between 6.9 months and 63.4 months (M = 27.4, SD = 13.2). The participants were recruited via existing participant databases and support groups. Ethical approval was obtained from the North West Wales Research Ethics Committee (13/WA/0194) and Birkbeck Ethics Committee (121373).

#### 2.1.2 Measure of vocabulary development

A parental questionnaire, the MacArthur-Bates Communicative Development Inventories: Words and Gestures version (CDI; Fenson et al., 2007) was used to collect data on their children’s receptive and expressive vocabularies. Because speech intelligibility problems can compromise estimates of productive vocabulary, parents were allowed to report both words and signs understood or produced as reflective of their vocabulary (see Yoder & Warren, 2004).

#### 2.1.3 Standardised measure of expressive and receptive language

Additional measures of expressive and receptive language development were collected by an experimenter during a lab visit. These were used to create a language composite score, with potentially lower measurement noise than the single CDI measure. The Mullen Scales of Early Learning (MSEL; Mullen, 1995) is a standardised assessment which comprises five domains: (1) *gross motor* (central motor control and mobility in supine, prone, sitting, and fully upright positions); (2) *fine motor* (visually-directed motor planning, object manipulation, visual discrimination, and motor control); (3) *visual reception* (visual perceptual ability, spatial awareness, and visual memory); (4) *receptive language* (auditory comprehension, auditory memory, and the ability to process linguistic input); and (5) *expressive language* (the ability to use sounds and language productively). MSEL is standardised for TD children between 0 and 68 months (from 0 to 33 months for gross motor domain). All participants scored under test ceiling on the MSEL language scales. The receptive and expressive language raw scores were normalised to the range 0 to 100, and averaged with the CDI scores of receptive and expressive vocabularies, similarly normalised, to create a language composite score. (See D’Souza et al., 2020, for a full description and analysis of the DS cohort’s developmental profile on the full MSEL battery).

#### 2.1.4 Measure of SES

SES was determined based on maternal and paternal occupations, using the highest major group for the two occupations as classified by the UK Office of National Statistics standard occupational classification 2010 (possible score range 1-9, with lower scores representing higher SES). Parental SES groups were: 1 managers / directors / senior officials, 2 professional occupations, 3 associate professional and technical occupations, 4 administrative and secretarial occupations, 5 skilled trade occupations, 6 caring, leisure and other service occupations, 7 sales and customer service occupations, 8 process, plant and machine operatives, 9 elementary occupations. The distribution of SES scores is shown in Table 1. SES data were missing for 5 families. The distribution of the SES scores was skewed, with most families falling in categories 1 to 4.

**Table 1:** Frequency distribution of SES scores for families of the infants and young children with DS, based on parental occupation (lower numbers = higher SES). SES data were missing for 5 families.

SES level								
1	2	3	4	5	6	7	8	9
24	31	13	4	2	3	1	1	0

*Note.* 1 = managers / directors / senior officials; 2 = professional occupations; 3 = associate professional and technical occupations; 4 = administrative and secretarial occupations; 5 = skilled trade occupations; 6 = caring, leisure and other service occupations; 7 = sales and customer service occupations; 8 = process, plant and machine operatives; 9 = elementary occupations.

### 2.2 Results

**Fig. 1:**
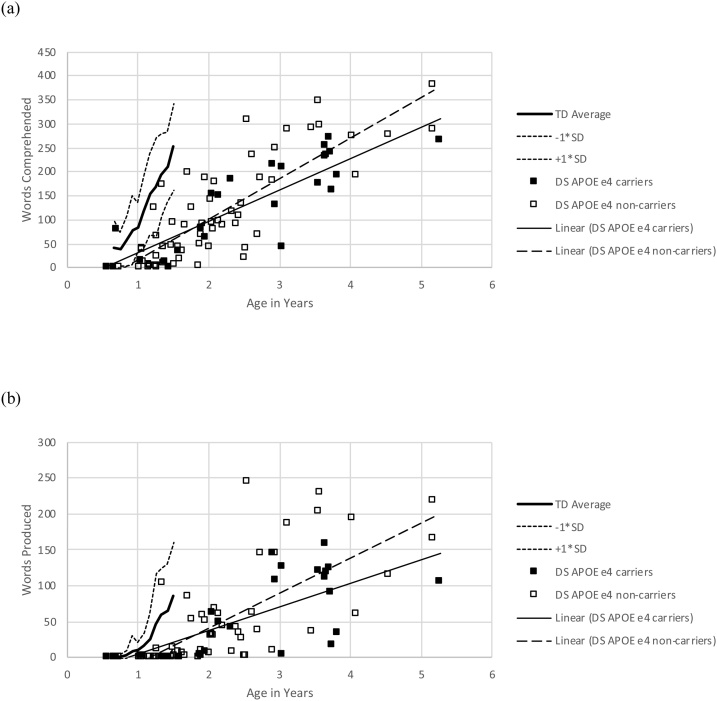
Parent ratings of (a) receptive and (b) expressive vocabulary sizes for children with DS, according to the CDI (Fenson et al., 2007). Children with DS are split by whether or not they are carriers of the *APOE* ε4 risk allele for dementia. Typically developing norms and standard deviations are also shown for 6-18 months of age (Fenson et al., 2007).

Parental ratings of their children’s receptive and expressive vocabulary sizes are shown in Fig. 1, which also includes TD norms and standard deviations from the standardisation sample from 6-18 months (Fenson et al., 2007). Comparison to the CDI TD norms demonstrates that, as expected, both receptive and expressive vocabulary were delayed compared to chronological age expectations. The relationship between receptive and expressive vocabulary appeared atypical in the DS sample, but not in the form of a greater deficit for expressive vocabulary (Martin et al., 2009). A linear regression equation predicting words produced from words comprehended was generated from the DS sample (R^2^ = .751, F(1,82) = 247.93, p < .001). The number of words produced was .57 (95% confidence intervals [CI]: .49, .64) times the number comprehended, with an intercept of -16.99 (CI: -28.32, -5.66). A similar regression from the CDI TD norms generated the equation: number of words produced = .40 X number comprehended – 20.98. Thus, the DS 95% confidence intervals overlapped with the TD intercept, but the gradient was higher for DS than that for TD (this was also the case when children with DS who were at floor on either measure were excluded). The higher than expected expressive vocabulary given the level receptive vocabulary may have arisen because parents were allowed to include manual signs, increasing estimates of expressive vocabulary.

There was large individual variability in the DS sample, evident in Fig. 1. For both receptive and expressive vocabulary, there were cases of children falling in the upper normal range for TD: one child was rated as having a receptive vocabulary of 81 words at 8.4 months, another as having an expressive vocabulary of 104 words at 16.2 months. There were also cases of older children with particularly delayed language: one child with a receptive vocabulary rated at 21 words at 30.2 months, another with an expressive vocabulary of 4 words at 36.6 months.

**Fig. 2:**
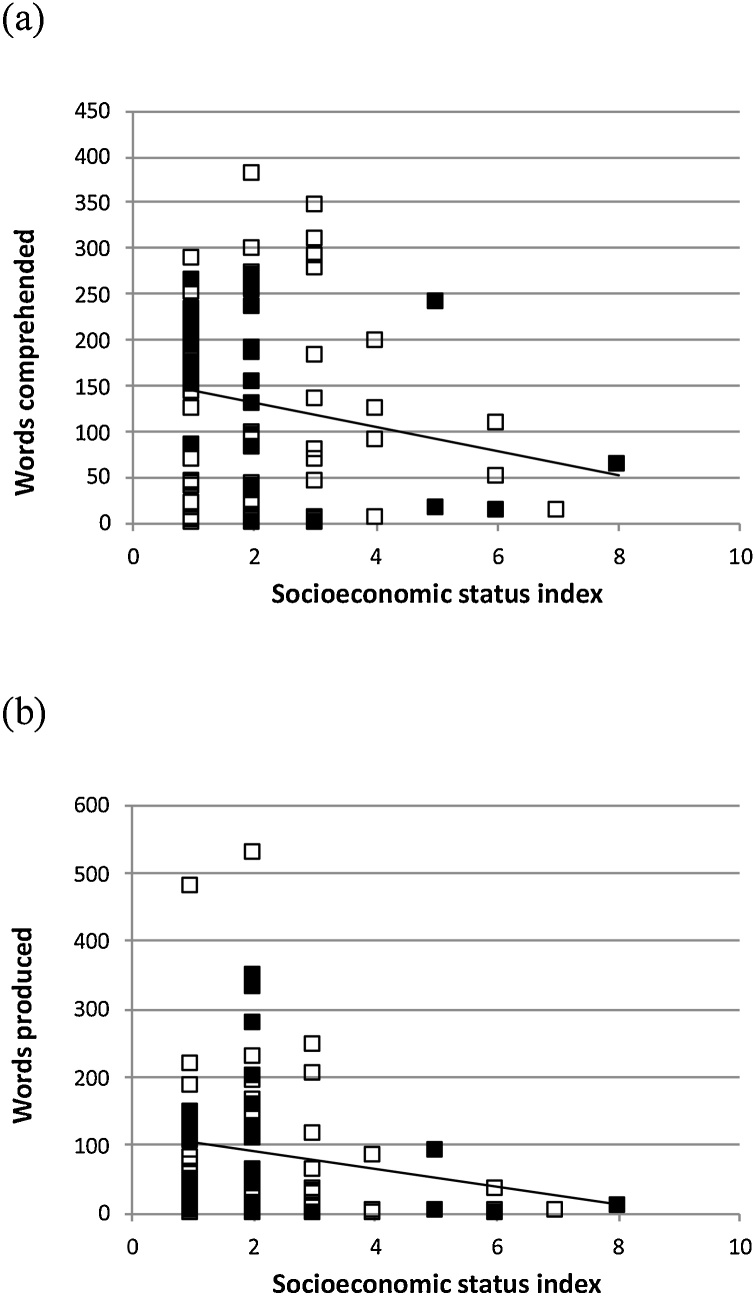
Parent ratings of (a) receptive and (b) expressive vocabulary sizes for children with DS according to the CDI (Fenson et al., 2007) plotted against SES, estimated by parental occupation (1 = highest SES, 9 = lowest SES). Children with DS are split by whether they are carriers of the *APOE* ε4 risk allele for dementia (filled markers) or non-carriers (non-filled markers).

Fig. 2 depicts the effects of SES on vocabulary size. These results are compromised somewhat by the uneven distribution of parental occupation categories across the sample. Although these plots are suggestive of higher vocabulary in higher SES families, the correlations are not reliable (receptive: r(79) = -.186, p = .101; expressive: r(79) = -.204, p = .071). Moreover, sampling produced a confound between SES and age; correlations partialling out chronological age were smaller still: receptive: r(76) = -.024, p = .837; expressive: r(76) = -.081, p = .478. When we examined the relationship between SES and our composite language measure, derived from combining receptive and expressive CDI scores and expressive and receptive scales from the MSEL standardised test, there was similarly no observed relationship with SES: r(78)= -.166, p = .146. There was therefore no evidence that SES, indexed by parental occupation, influenced children’s vocabulary size in this sample.

### 2.3 Discussion

The current data, from a reasonably sized sample of infants and young children with DS compared to the literature, confirmed extensive individual differences in both receptive and expressive vocabulary. It is possible that some of the variance arises from limitations / noise in the CDI measure itself, though one would need to explain why this noise was larger for parental ratings of children with DS than those of TD children. Perhaps this could be due to difficulties in discerning poor articulation or the use of gestures in some children with DS. The relationship between receptive and expressive vocabulary showed a higher expressive vocabulary given receptive vocabulary than would be expected from the CDI norms. This differs from patterns reported elsewhere, of an expressive vocabulary deficit (Martin et al., 2009, for review) and may have arisen because parents were allowed to include manual signs, increasing estimates of expressive vocabulary.

SES did not predict a reliable amount of the variation in vocabulary reported for our sample, or in a composite measure, perhaps more robust to measurement error, generated by combining CDI and MSEL scores. SES is a strong predictor of individual differences in vocabulary in typical development. Fernald, Marchman and Weisleder (2013) observed correlations of around 0.3 in 18-24 month olds. That SES was not predictive in the current sample suggests that the DS genotype is the overriding influence. We should note that in their study of 48 3-year-olds with DS, Deckers et al. (2017) reported age-corrected correlations of maternal education to expressive vocabulary of around 0.5, though curiously no effect was observed on receptive vocabulary. The disparity may arise due to the different sampling of SES ranges, so that SES is a reliable predictor where families with lower SES are included.

We now turn to consider whether a single genetic factor, *APOE* genotype, was successful in predicting the observed within-DS-group variation.

## 3 Influence of a single genetic factor, *APOE* genotype, on early vocabulary development in DS

### 3.1 Method

#### 3.1.1 Participants

The participants were the same as the previous study.

#### 3.1.2 Establishing APOE genotype

*APOE* genotype was determined using a Thermo Fisher Scientific Taqman assay for SNPs rs7412 and rs429358 (Waltham, MA, USA). Children were categorised according to whether they carried the ε4 risk allele (either heterozygously or homozygously) or did not carry the ε4 allele (ε2 and ε3 genotypes were not distinguished). There were 25 ε4 carriers (14 female, 11 male) and 59 non-carriers (37 male, 22 female), a proportion that reflects the distribution in the typical population (van der Lee et al., 2018). The *APOE* groups did not differ in key participant characteristics (see Table 2).

**Table 2:** Participant characteristics for infants and toddlers with DS split by *APOE* status

		*e4*-carriers	*e4*-noncarriers	Comparison *e4*-carriers vs. *e4*-noncarriers
Number		25	59	N/A*
Age		7-63 months *M* = 31, *SD* = 14.8	7-62 months *M* = 26 *SD* = 12.2	*t*(82) = 1.61, *p* = .112
*APOE* genotype	*e2/2*	0 (0.0%)	0 (0.0%)	N/A*
	*e2/3*	0 (0.0%)	12 (20.3%)	
	*e3/3*	0 (0.0%)	47 (79.7%)	
	*e2/4*	1 (4.0%)	0 (0.0%)	
	*e3/4*	22 (88.0%)	0 (0.0%)	
	*e4/4*	2 (8.0%)	0 (0.0%)	
Gender	Female	14 (56.0%)	22 (37.3%)	*χ^2^*(1) = 2.51, *p* = .113
	Male	11 (44.0%)	37 (62.7%)	
Ethnicity	White	23 (92.0%)	47 (79.7%)	Fisher’s exact test = 2.68, *p* = .675
	Asian	0 (0.0%)	5 (8.5%)	
	Black	1 (4.0%)	3 (5.1%)	
	Mixed	1 (4.0%)	3 (5.1%)	
	Other	0 (0.0%)	1 (1.7%)	

N/A = not applicable

* The prevalence of *APOE* genotypes reflects the distribution in the general population (van der Lee et al., 2018).

### 3.2 Results

Separate fully factorial analyses of covariance (ANCOVAs) were used to assess the role of *APOE* genotype on the CDI measures of parent-rated receptive and expressive vocabulary development, with between-subjects factors of *APOE* genotype and gender, and the covariate of chronological age in days (Thomas et al., 2009). Linear trajectories of vocabulary growth split by *APOE* group (ε4 carriers versus non-carriers) are included in Fig. 1. Both vocabulary measures indicated a reliable increase in vocabulary size with chronological age (receptive: F(1,76) = 154.81, p < .001, η_p_^2^ = .671; excluding participants at floor which may flatten the developmental trajectory: F(1,71) = 76.57, p < .001, η_p_^2^ = .519; expressive: F(1,76) = 72.37, p < .001, η_p_^2^ = .488; excluding floor: F(1,71) = 35.00, p < .001, η_p_^2^ = .330). There was no reliable effect of *APOE* genotype or interaction with age (receptive, main effect: F(1,71) = .75, p = .389, η_p_^2^ = .010; interaction with age: F(1,76) = 2.02, p = .159, η_p_^2^ = .026; excluding floor: main effect F(1,71) = 1.31, p = .256, η_p_^2^ = .018, interaction F(1,71) = 2.40, p = .126, η_p_^2^ = .033; expressive, main effect: F(1,76) = .78, p = .379, η_p_^2^ = .010; interaction with age; F(1,76) = .519, p = .473, η_p_^2^ = .007; excluding floor: main effect F(1,71) = 1.20, p = .277, η_p_^2^ = .017, interaction F(1,71) = 2.16, p = .146, η_p_^2^ = .030). There were no reliable main effects of gender (receptive: F(1,76) = .33, p = .569, η_p_^2^ = .004 ; expressive: F(1,76) = .65, p = .423, η_p_^2^ = .008) or interactions.

**Fig. 3:**
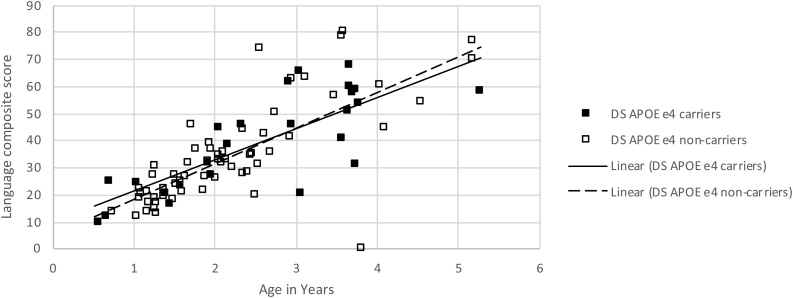
Composite language measure for the children with DS, derived from CDI (Fenson et al., 2007) and MSEL receptive and expressive language measures (Mullen, 1995) plotted against age. Children with DS are split by whether or not they are carriers of the *APOE* ε4 risk allele for dementia.

We carried out a similar fully factorial ANCOVA assessing the effect of *APOE* genotype on our language composite measure, which was potentially more robust to measurement noise (Fig. 3). There was again no reliable effect of *APOE* genotype or interaction with age (main effect: F(1,80) = 1.49, p = .226, η_p_^2^ = .018; interaction with age: F(1,80) = 2.17, p = .145, η_p_^2^ = .026). Finally, we entered SES into the model as an additional co-variate. SES showed no reliable main effect or interactions. In this more complex model, the main effect of *APOE* showed a trend (F(1,68) = 3.68, p = .059, η_p_^2^ = .051), with higher scores for ε4 carriers compared to non-carriers, and there was also a trend for an interaction between *APOE* and age, with ε4 carriers scoring higher than non-carriers at younger ages and lower at older ages (F(1,68) = 3.60, p = .062, η_p_^2^ = .050).

### 3.3 Discussion

*APOE* genotype was deemed a possible predictor of individual differences since in adulthood in DS, it increases the risk of AD by a hazard ratio of 1.8 and there is evidence that the genotype also influences early brain development (Dean et al., 2014; Prasher et al., 2008). Here, *APOE* genotype did not account for statistically reliable amounts of the wide individual differences observed in vocabulary acquisition. One might note that the study, despite a reasonable sample size, was underpowered to detect a difference that was comparable to the hazard ratio for AD in DS associated by carriers of *APOE* ε4. Converting the hazard ratio of 1.8 into a η_p_^2^ equivalent of .026 (Borenstein et al., 2009; Cohen, 1988; see DeCoster, 2012, for calculation), *a priori* power analyses suggest a sample approximately 3 times larger would be necessary to detect the effect on vocabulary in the ANCOVA design with 80% power assuming this equivalent effect size.

However, such an *a priori* power analysis would necessarily assume that the *APOE* effect size was consistent across lifespan. Recently, in a study focusing on on-line measures of selective and sustained attention, it has been reported that ε4 carrier status may confer an *advantage* in early development, which diminishes across childhood, and only later is associated with a disadvantage linked to cognitive decline (D’Souza, Mason et al., 2020). It is notable that the final analysis of *APOE* effects on the language composite, which included both age and SES as covariates, yielded the same pattern of early ε4 advantage. However, this was only present as a statistical trend, which would be weakened further by any Bonferroni corrections for multiple comparisons / analyses.

In sum, the main finding was that for this particular phenotypic measure, vocabulary development, a single gene variant that displays marked involvement in the DS phenotype during ageing was not sufficient to account for much of the wide individual variation that results from the multitude of genes whose expression is altered by trisomy of chromosome 21. An alternative avenue to predict those individual differences, however, is to assess the net effect of all the changes in genes expression on brain development at a very young age, rather than isolate individual genes. As we show in the next section, advances in neuroimaging now allow for structural MRI of prenatal and neonatal brains in DS. The challenge is to assess whether these measures can serve as a link to individual differences in infant and child cognitive development. We assess the viability of this enterprise in the next section.

## 4 Proof of concept: predicting variations in the cognitive development of infants with DS from prenatal and neonatal measures of brain structure

There is substantial published evidence from mouse model studies (e.g., Aziz et al., 2018), histological studies (e.g., Olmos-Serrano et al., 2016) and human neuroimaging studies (e.g., Carducci et al., 2013; Pujol et al., 2015; Śmigielska-Kuzia et al., 2011) demonstrating abnormal brain development, structure and functioning in DS. Recent evidence has shown that, in humans with DS, those brain abnormalities such as reduced brain volume are present before birth (Patkee et al., 2019). Deviations in brain growth start from 22 weeks gestation in the fetus with DS, showing significant cerebellar, cortex and brain volume reductions by term age (Patkee et al., 2019).

These brain abnormalities are likely to reflect the biological correlates of the intellectual disability (Carducci et al., 2013; Śmigielska-Kuzia et al., 2011; Dierssen, 2012) and delayed development present in children with DS. Recent developments in antenatal neuroimaging techniques, such as MRI, now allow accurate and precise quantification of both structure and function within the fetal and the neonatal brain (Ferrazzi et al., 2014; Jiang et al., 2009; Malamateniou et al., 2013) and it is also feasible to extend these methods to DS (Baburamani et al., 2019). This presents an ideal opportunity to examine whether early brain biomarkers known to be abnormal in the DS population (e.g., cortical or cerebellar volumes) are predictive of later cognitive outcomes, and indeed might be able to provide *in vivo* surrogate outcome measure for future interventions. We have begun pilot work to assess this possibility.

### 4.1 Method

#### 4.1.1 Participants

Over the last five years, fetuses and neonates with DS have been recruited with ethical approval at the Centre of the Developing Brain at St. Thomas’ Hospital, London, UK. Data from this large sample are presented in Patkee et al. (2019). To date, five of these infants were followed up for cognitive testing at Birkbeck’s Centre for Brain and Cognitive Development. At testing, these children were aged between 6 and 17 months. Data from the five case studies were compared with data from two larger DS groups, one contributing brain data, one contributing behavioural data (brain volume: Patkee et al., 2019; behavioural data for MSEL: D’Souza, Karmiloff-Smith et al., 2020). TD cross-sectional growth trajectories were then plotted. For brain volume, the comparison groups comprised 45 scans from individuals with DS, age 21 weeks gestation to 46 weeks postmenstrual age (PMA)^3^ (M = 34.46 weeks; SD = 6.92), and 40 TD individuals, age 30 weeks gestational age to 44 weeks PMA (M = 37.24 weeks; SD = 4.71). For behaviour, the comparison groups comprised 99 individuals with DS, age 6-63 months (M = 29.55; SD = 13.98) and 62 TD individuals from the LonDownS cohort, age 4-53 months (M = 23.60 months; SD = 11.33). The behavioural data came from a sample that overlapped with that reported in studies 1 and 2.

#### 4.1.2 Magnetic Resonance Imaging

**Fig. 4:**
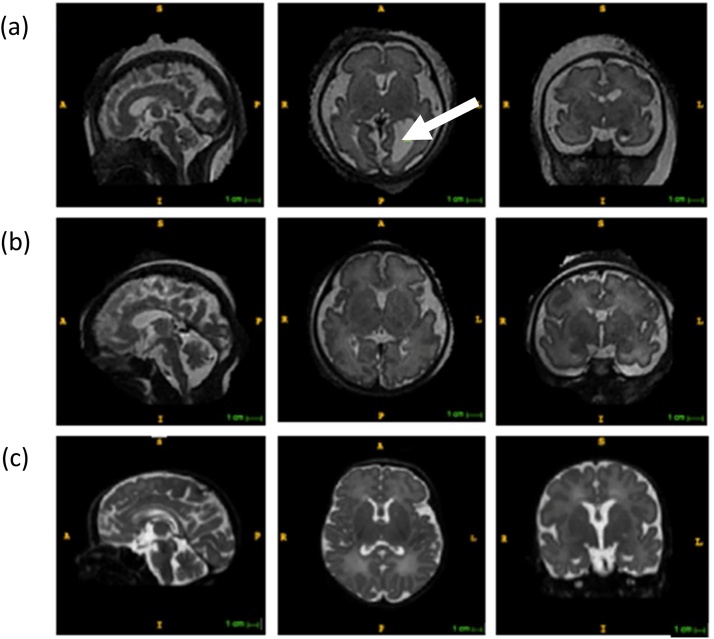
Structural MRI images of fetuses and a neonate with DS after motion correction and reconstruction (SVR) (left to right: sagittal, axial and coronal planes). (a) 30-week-old fetus also demonstrating unilateral ventriculomegaly, enlargement of the ventricles of the brain (shown by arrow); (b) 34 week old fetus; and (c) 43 week old neonate.

MRI scans were acquired at 1.5 T or 3 T from 20 weeks gestation to 46 weeks PMA. T2-weighted images were acquired in the sagittal and transverse planes using a multi-slice turbo spin echo sequence. Examples of images that can be acquired are shown in Fig. 4. Post-acquisition processing of the raw imaging data used a process known as snapshot volume reconstruction (SVR) (Jiang et al., 2007) to generate 3D reconstructions and volumetric segmentations of several anatomical regions were then performed using both semiautomatic and manual processes. These included measurement of the volumes of the supratentorial brain (the brain area above the cerebellar tentorium, containing the cerebrum, and excluding the cerebellum), the cortex and the cerebellum. See Patkee et al. (2019) for further details of MRI methods.

#### 4.1.3 Measures of cognitive development

The Mullen Scales of Early Learning (MSEL) (Mullen, 1995) were used to provide a standardised developmental measure of cognitive functioning. Scores were obtained on five scales: the gross motor scale, visual reception, fine motor abilities, receptive and expressive language.

### 4.2 Results

**Fig. 5:**
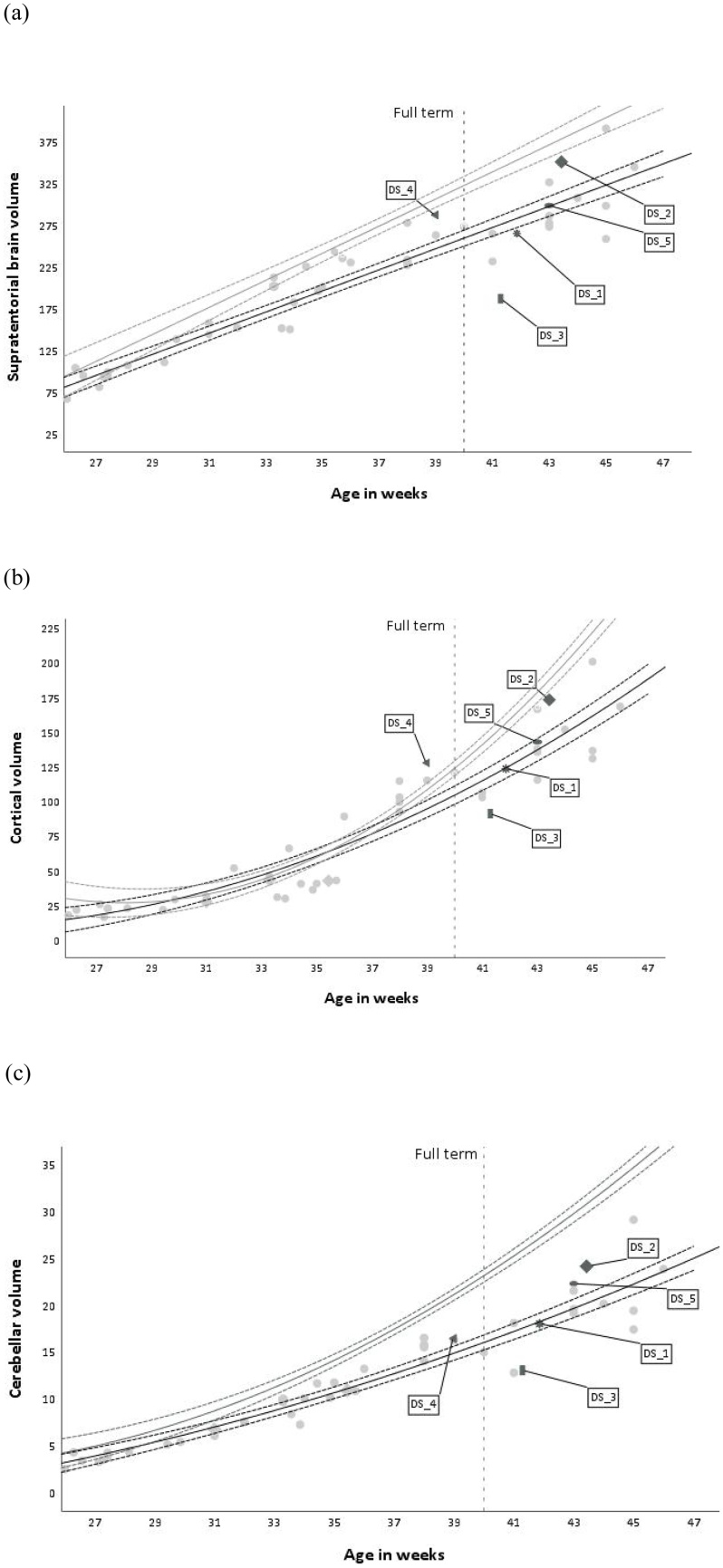
(a) Supratentorial brain volume (cm^3^), (b) cortex volume (cm^3^), and (c) cerebellar volume (cm^3^) changes with age in a sample of fetuses and neonates with DS (age 22 weeks GA to 46 PMA). Labels are attached to those neonatal cases who were later assessed with the MSEL. TD cross-sectional growth trajectory and 95% CI shown in grey for comparison (DS and TD trajectories were generated from data in Patkee et al., 2019). The dotted vertical line shows usual full term (though babies may be born prematurely).

**Fig. 6:**
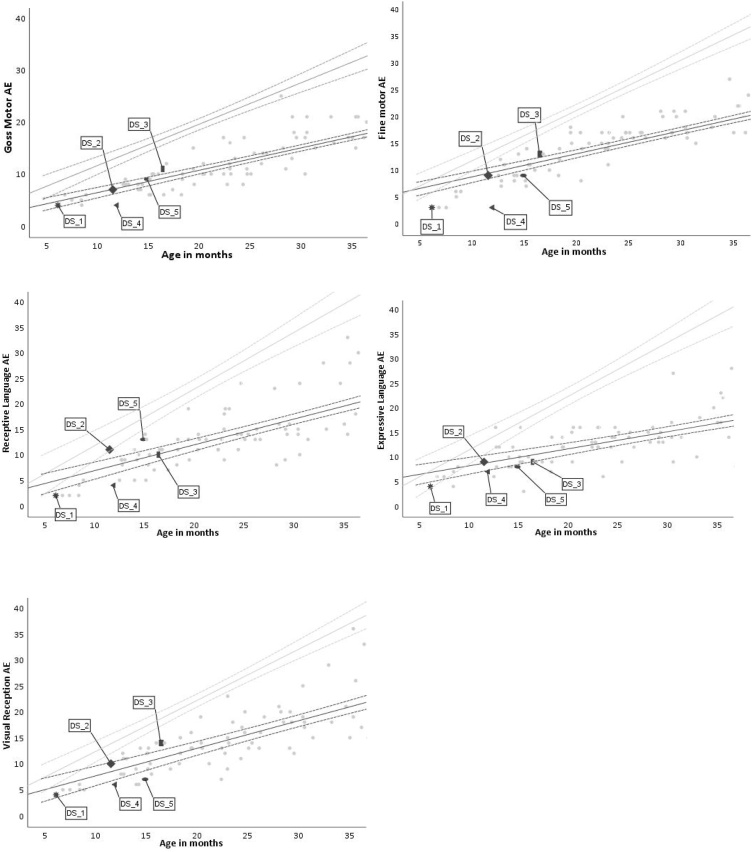
MSEL age-equivalent (AE) score changes with age in a sample of infants and children with DS for the subtests of gross motor, fine motor, visual reception, receptive language and expressive language. Labels are attached to those cases who had an MRI scan in the perinatal period. TD cross-sectional growth trajectory and 95% CI shown in grey for comparison (DS and TD group data taken from D’Souza et al., 2020).

In order to contextualise the results for the five children with DS, we plot their MRI data against the larger cohort of fetuses and neonates with DS reported in Patkee et al. (2019), and their MSEL behavioural profiles against the larger sample of infants and children with DS tested as part of the LonDownS cohort (D’Souza et al., 2020). Fig. 5 shows the supratentorial, cortical and cerebellar volumetric changes related to age. It demonstrates little individual variability in brain volumes earlier in the fetal period (up to approximately 32 weeks gestation) within the larger group, and how these become more pronounced with increasing age. Fig. 6 show the changes in MSEL related to chronological age in participants with DS. Since the goal was to consider the position of each child with respect to other individuals with DS, we generated standardised residuals against the DS trajectory for the three brain measures and five behavioural measures. The standardised brain residuals were generated from regressing MRI regional volumes on age from the 45 individuals with DS in the Patkee et al. (2019) sample. The standardised behavioural residuals were generated from regressing scores on the five MSEL sub-scales on age from the 99 individuals with DS in the D’Souza, Karmiloff-Smith et al. (2020) sample. Table 3 indicates how far above or below each child fell compared to the group trajectory for their age at testing. We could then assess the relationship between early MRI derived brain region volumes and subsequent cognitive outcomes.

**Table 3:** Standardised residuals for each of the 5 DS case studies for all neonatal brain volume measures (supratentorial, cortical and cerebellar) and MSEL scales of infant cognitive development.

Case ID	Brain volumes			MSEL				
	Supratentorial brain	Cortex	Cerebellum	GM	FM	VR	RL	EL
DS_1	-.657	.013	-.185	-.239	−1.233	-.273	-.557	-.541
DS_2	1.671	1.790	1.922	-.022	-.122	.262	.645	.053
DS_3	−3.163	−1.299	−2.208	.584	.449	.500	-.023	-.269
DS_4	1.400	1.189	.391	−1.038	−1.989	-.457	-.737	-.353
DS_5	.013	.522	1.271	.154	-.561	-.555	.701	-.358

*Note.* MSEL = Mullen Scales of Early Learning; GM = gross motor; FM = fine motor; VR = visual reception; RL = receptive language; EL = expressive language.

Comparison of neonatal brain volume and MSEL score plots case by case suggest different patterns of relationship. The participant labelled DS_1 had below average brain volumes, which matched to below average scores in all MSEL scores at 6 months old. A similar consistent pattern was found in the participant labelled DS_2, but in this case, all values were above or around the group’s mean. However, this pattern did not hold in the other three cases, where being below the group mean for brain volume did not match to being below the group average in the cognitive outcome, and vice versa. In this small sample, therefore, the results were inconclusive.

### 4.3 Discussion

The pilot data presented here demonstrate the viability of collecting the data to evaluate whether differences in neonatal brain volumes in DS are predictive of early cognitive development, but initial data are too few to draw conclusions, and work is still on going. It does appear possible to test the relationship between perinatal brain volumes and childhood cognitive outcome in children with DS, both of which reflect the net genetic effect of the DS mutation. For example, one could test the hypothesis that specific abnormalities in the cerebellum might predict subsequent motor deficits, or that cortical volumes would predict overall IQ or core cognitive measures such as language. Should such relationships arise, the mechanisms by which brain volumes relate to cognitive outcomes would need to be further established, as this may be driven by several factors, such as volumes of cellular bodies, neural synapses, white matter tracts or even extracellular matrix.

## 5 Conclusion

We focused on individual differences in DS – rather than differences compared to typically developing controls – seeking to integrate causes across multiple levels including genes, brain, behaviour, and environment. We also sought to conceptualise individual differences in a lifespan developmental framework, seeking potential links between the marked variation observed in early development and the elevated risk for dementia in adulthood. We asked whether it was possible to identify already in infancy and early childhood in this very high-risk population, risk or protective factors for later AD (Karmiloff-Smith, 2016; Karmiloff-Smith et al., 2016). Such a mapping is much harder to test in typical development, since the incidence of AD is lower and we do not know which children will go on to develop AD, whereas it is known that in DS all individuals will show the histopathology of the brain linked with AD by middle adulthood (Head, Powell et al., 2012).

Beginning at the environmental level, we investigated whether SES would demonstrate the predictive power for variations in rate of vocabulary development that is observed in typical development (e.g., Fernald et al., 2013). Karmiloff-Smith (2016) speculated that SES may play a more serious role in DS, based on evidence of early effects of SES on infant brain development (Tomalski et al., 2013), and the possibility that low SES may constrain the parents’ opportunities to support the development of their child. The mechanisms by which SES exerts its impact on development are various and may depend on the population in question, but involve at least three pathways: prenatal influences (such as maternal diet and stress), postnatal nurturing, and postnatal cognitive stimulation (Hackman et al., 2010). For typical vocabulary development, studies suggest that the pathway largely involves cognitive stimulation, depending on the density of the language use and communicative interactions of the parents with the child (Hoff, 2003). In a sample of 84 infants and young children with DS, we found wide individual differences but very little of this variation was predicted by SES (as assessed by parental occupation), amounting to 4% at most, and not statistically reliable. However, our SES range was somewhat restricted, with fewer families from lower SES groups. Other studies have reported reliable effects of SES on DS vocabulary (e.g., Deckers et al., 2017), and there is evidence that vocabulary development in children with intellectual disabilities including DS is partly predicted by how parents respond to their children’s communication (Yoder & Warren, 2004). There is also evidence that early in development, mothers of children with DS alter their vocabulary to support noun learning (Kay-Raining Bird & Cleave, 2016). Deckers (2017) argued that mothers of children with a higher educational level are more able to fine-tune their talk to their child to support vocabulary learning. But based on his own data, Deckers reported such scaffolding fades out as the children develop better communicative abilities.

Nevertheless, our data suggest any such effects are weak, with the causes of DS variability either generated by the genotype or by environmental measures not indexed by parental occupation – that is, *even though SES was not a reliable predictor in our data, there was extensive individual variability in vocabulary development*. While our CDI data likely included measurement noise, SES predicted no more of the variance in a composite language measure that should have been more robust to noise.

Lastly, connecting with the lifespan developmental perspective, we note that Startin et al. (2020) recently identified SES, indexed by parental occupation, as a reliable predictor of variation in cognitive ability in young adults with DS. These authors viewed the effect as a soft marker for the wider educational, social, and therapeutic opportunities that can support cognitive skills into adulthood. We also note that SES has been identified as a reliable predictor of dementia in the general population, where Cadar et al. (2018) reported the hazard of developing dementia to be 1.68 times higher for those in the lowest wealth quintile compared with those in the highest quintile, independent of education, index of multiple deprivation, and health indicators. We are not aware of any data that identifies the effect of SES on dementia risk for adults with DS, though living status (whether the individual was living with their family or not) was a reliable predictor (Sinai et al., 2018). However, SES may not have the same meaning for adults with DS, who are often living in supported circumstances. And cross-sectional comparisons of environmental effects in infants and adults may be subject to cohort effects.

Turning to the genetic level, we took a known genetic risk factor for dementia in DS in adulthood, the *APOE* genotype, and assessed whether it explained any of the variation we observed in early vocabulary development. There are at least three ways that early development and adult resilience to AD pathology may be linked: (1) development and pathological ageing may involve separate mechanisms, so that individual differences in the two would be independent; (2) development may contribute to resilience – in the form of *brain reserve* (the amount of brain capacity that can be lost before damage manifests in behaviour, often indexed by intracranial volume) or in the form of *cognitive reserve* (cognitive compensatory mechanisms to deliver behaviour despite damage, often indexed by educational attainment) (Groot et al., 2018) – but otherwise development variation would be separate from mechanisms of ageing; (3) variation in development may be linked to variation in ageing by shared mechanisms, with two possibilities: (a) the mechanisms that make the system less efficient at development may also make it more vulnerable to ageing; or conversely, (b) the mechanisms may have opposing effects, so that systems more vulnerable to ageing may show faster early development, perhaps because they are more hurriedly but less robustly assembled; perhaps because the biological mechanism that confers some mechanical or functional benefit also stresses the system more in the longer term; perhaps because lifespan development as a whole is accelerated so that individuals show both faster development and faster ageing. Given the complex dynamics of cognitive development, such possibilities of course need much clarification, particularly with respect to whether the effects would be expected to be specific to cognitive domains or skills, or be general across cognition.

With respect to vocabulary development, our results showed no overall reliable effects of *APOE* genotype, so that children with the risk ε4 allele (N = 25) did not show different levels or rates of vocabulary development than those who were not carriers (N = 59). It is possible the sample was underpowered to detect a small effect size, so that the null result was therefore insufficient to distinguish the possibilities at this stage. The strongest effects were observed in the language composite measure, which combined parental ratings of vocabulary size with lab-based standardised assessments of receptive and expressive language skills. For this measure, a trend was observed towards an advantage for ε4 carriers that reduced with age (main effect of *APOE* group p = .059, interaction with age p = .062). The fact that differences have been observed in the brain development of typically developing infant ε4 carriers compared to non-carriers (Dean et al., 2014) implicates common mechanisms linking development and ageing, and therefore the third possibility. Data on early visuo-attentional skills of the same cohort reported in study 2 found an attentional advantage for the DS ε4 carriers over non-carriers which reduced with age (D’Souza, Mason et al., 2020), similar to the trend pattern above. If these effects are real, they support 3(b). This would align with a recent review of the role of the ε4 *APOE* genotype in young carriers with respect to cognition, where Smith, Ashford and Perfetti (2019) argued that ε4 is associated with improved fitness during fetal development, infancy, and youth relative to the ε3 allele, at the expense of decreased fitness in old age; and proposed that a putative mechanism is an elevated level of synaptic macromolecular turnover, which then stresses basic cellular neuroplasticity mechanisms, rendering them subsequently more vulnerable.

The small predictive power of individual gene variants led us to consider the brain level, and in particular brain development as the locus of the concerted action of genetic variations in DS (full trisomy versus partial versus mosaic; variations in genes on chromosome 21; interactions with genetic variation on other chromosomes, including epigenetic regulation of gene expression). Recent data suggest that in DS, divergence from typical patterns of prenatal brain development can be observed as early as 22 weeks gestation (Patkee et al., 2019), although again there is variability, and differences from TD are not always apparent. Latest methods in the structural brain imaging of fetuses and neonates mean it may be feasible to correlate variation the size of macro brain structures to subsequent infant cognitive development. Once more, the focus is not to note differences compared to TD, but within the DS cohort itself. In the five case studies we have so far tracked, we did not observe a consistent pattern, and a larger sample is necessary to evaluate whether developmental links between variations in brain and behaviour can be uncovered. We have established that the method is viable, and the potential exists to use early brain imaging as a marker for future outcomes to guide clinical interventions. They may also provide a locus to explore the effects of specific genes on regional brain development, such as the *APOE* genotype considered here. However, from a theoretical point of view, we should also recognise that coarse macro measures of brain structure may give limited insights into the key properties influencing behaviour. More sensitive measures are desirable, such as those of brain connectivity that can be delivered by diffusion MRI and tractography (Baburamani et al., 2019). This work is also currently underway. Lastly, some key properties of individual variation at the brain level may lie at the micro-scale, at the levels of neurons and neurotransmitters, such as in the relative balance between excitatory and inhibitory neural activity (suggested by animal models, e.g., Kleschevnikov et al., 2004). This will require functional imaging methods to investigate.

### 5.1 The contribution of Annette Karmiloff-Smith

The research reported here was inspired by the theoretical framework of Annette Karmiloff-Smith, both in its emphasis on a multi-level, multi-method approach and its commitment to placing developmental mechanisms at the heart of explanations of disorders and variability (*neuroconstructivism*; Karmiloff-Smith, 1998, 2009; Karmiloff-Smith et al., 2012; Thomas & Karmiloff-Smith, 2002). It was the lifespan developmental perspective that led her to argue that dementia should itself be seen as a developmental disorder (Karmiloff-Smith et al., 2016); and to her involvement in the LonDownS project that sought to explore variation and possible sub-groups in a cohort of over 100 children with DS by simultaneously considering behaviour (adaptive behaviours, audio-visual integration, visuo-attention, memory, deferred imitation, motor skills, language, social engagement), brain structure and function, genotype (including AD risk), sleep, parental IQ, parent-child interaction, and SES, in cross-sectional and longitudinal studies. The origin of individual differences in DS is likely to be complex, and Karmiloff-Smith’s approach was not to simplify but to embrace this complexity. This paper contains arguments and speculations that Annette made in her last presentation of this project, for example regarding the potential links between infancy and dementia in DS, and the role of SES (Karmiloff-Smith, 2016). Work continues in her memory, following up this infant cohort to explore the consistency of individual differences through childhood.

## CRediT authorship contribution statement

**Michael S.C. Thomas:** Conceptualization, Formal analysis, Funding acquisition, Investigation, Methodology, Supervision, Writing - original draft. **Olatz Ojinaga Alfageme:** Conceptualization, Data curation, Formal analysis, Investigation, Methodology, Project administration, Visualization, Writing - original draft. **Hana D’Souza:** Conceptualization, Data curation, Funding acquisition, Investigation, Project administration, Supervision, Writing - review & editing. **Prachi A. Patkee:** Conceptualization, Data curation, Formal analysis, Methodology, Project administration, Visualization. **Mary A. Rutherford:** Conceptualization, Funding acquisition, Investigation, Methodology, Supervision, Writing - review & editing. **Kin Y. Mok:** Data curation, Formal analysis, Methodology, Validation. **John Hardy:** Conceptualization, Funding acquisition, Investigation, Methodology. **the LonDownS Consortium:** Conceptualization, Data curation, Formal analysis, Funding acquisition, Investigation, Methodology, Project administration, Resources, Software, Supervision, Validation, Visualization, Writing - original draft, Writing - review & editing. **Annette Karmiloff-Smith:** Conceptualization, Funding acquisition, Investigation, Methodology, Resources, Supervision.

## Footnotes

3 Gestational age (GA) is a measure of the age of a baby during pregnancy, which is based on the beginning of the woman's last menstrual period, or on the earliest antenatal ultrasound. Postmenstrual age (PMA) is a measure of the age of a baby in weeks from birth. A preterm baby born at 35 weeks GA will be 36 weeks PMA one week after birth.
